# Improved health status with insulin degludec compared with insulin glargine in people with Type 1 diabetes

**DOI:** 10.1111/j.1464-5491.2011.03547.x

**Published:** 2012-06

**Authors:** P D Home, L Meneghini, U Wendisch, R E Ratner, T Johansen, T E Christensen, J Jendle, A P Roberts, K I Birkeland

**Affiliations:** 1 Institute of Cellular Medicine—Diabetes, Newcastle UniversityNewcastle upon Tyne, UK; 2 Miller School of Medicine, University of MiamiMiami, FL, USA; 3 Gemeinschaftspraxis für Innere Medizin und DiabetologieHamburg, Germany; 4 MedStar Health Research InstituteHyattsville, MD, USA; 5 Novo Nordisk A/SSøborg, Denmark; 6 Endocrine and Diabetes Centre, Karlstad Hospital, Faculty of Health Sciences, Örebro UniversityÖrebro, Sweden; 7 Endocrine and Metabolic Unit, Royal Adelaide HospitalAdelaide, SA, Australia; 8 Faculty of Medicine, University of Oslo, Department of Endocrinology, Oslo University HospitalOslo, Norway

**Keywords:** hypoglycaemia, insulin, insulin therapy, quality of life, Type 1 diabetes

## Abstract

**Aims:**

The efficacy and safety of insulin degludec (degludec), a new-generation ultra-long-acting basal insulin, was compared with insulin glargine (glargine) in people with Type 1 diabetes mellitus in a 16-week, open-label, randomized trial. Health status, an important aspect of effective diabetes management, was also assessed.

**Methods:**

Degludec (*n* = 59) or glargine (*n* = 59) were injected once daily, with insulin aspart at mealtimes. Health status assessment utilized the validated Short Form 36 Health Survey, version 2, which has two summary component scores for mental and physical well-being, each comprising four domains.

**Results:**

At study end, HbA_1c_ reductions were comparable between groups, but confirmed nocturnal hypoglycaemia was significantly less frequent with degludec [relative rate 0.42 (95% CI 0.25–0.69)], and overall hypoglycaemia numerically less frequent [relative rate 0.72 (95% CI 0.52–1.00)]. After 16 weeks, a significant improvement in Short Form 36 Health Survey mental component score of +3.01 (95% CI 0.32–5.70) was obtained for degludec against glargine, attributable to significant differences in the social functioning [+8.04 (95% CI 1.89–14.18)] and mental health domains [+2.46 (95% CI 0.10–4.82)]. For mental component score, Cohen’s effect size was 0.42, indicating a small-to-medium clinically meaningful difference. The physical component score [+0.66 (95% CI –2.30 to 3.62)] and remaining domains were not significantly different between degludec and glargine.

**Conclusions:**

In the context of comparable overall glycaemic control with glargine, degludec improved mental well-being as measured using the mental component score of the Short Form 36 Health Survey. The improvements in overall mental component score and the underlying social functioning and mental health domains with degludec compared with glargine may relate to the observed reduction in hypoglycaemic events.

## Introduction

Effective diabetes management is dependent upon establishing good glycaemic control and improving the way patients perceive their treatment regimen [1, 2]. There is clear evidence that diabetes can negatively impact health-related quality of life. Studies have identified reasons including, but not limited to, regimen complexity, fear of injections and fear of hypoglycaemia [1, 3–9]. Accordingly, professional association guidance recommends that, to maintain treatment adherence and health-related quality of life, hypoglycaemia should be avoided where possible [10]. Therapeutic approaches that provide a stable glucose profile, without peaks and troughs, therefore appear desirable. Moreover, as global healthcare budgets tighten, assessing health-related quality of life and health status to facilitate cost-effectiveness decisions is becoming increasingly important.

Insulin degludec (degludec) is a new-generation basal insulin, designed to form soluble multi-hexamers on subcutaneous injection, resulting in an ultra-long action profile [11] and reduced within-individual variability [12]. The efficacy and safety of degludec compared with insulin glargine (glargine) in people with Type 1 diabetes mellitus have been reported in a 16-week, open-label, randomized study [13]. This pre-specified analysis investigates the effect of degludec vs. glargine on health status during the trial period, using a validated generic questionnaire [Short Form 36 Health Survey (SF-36) version 2; QualityMetric Inc., Lincoln, RI, USA] designed to specifically assess health status [14].

## Patients and methods

A more detailed description of the study population, baseline characteristics, study methodology and metabolic outcomes is given in the publication of Birkeland *et al*. [13].

### Study population and baseline characteristics

Briefly, eligible participants aged 18–75 years, with Type 1 diabetes, using any insulin regimen, and with HbA_1c_ 53–97 mmol/mol (7.0–11.0%) were enrolled and randomized to glargine 100 U/ml (*n* = 59) or degludec 100 U/ml (*n* = 59), both combined with mealtime insulin aspart (100 U/ml), for a treatment period of 16 weeks. A third arm with degludec in an alternative formulation was also included. However, as clinical development of this formulation has been discontinued, it was excluded from this analysis. At baseline, participants had a mean age of 45.8 years, mean HbA_1c_ of 68 mmol/mol (8.4%) and mean body mass index of 26.9 kg/m^2^. Both basal insulin products were administered once daily in the evening, preferably at the same time each day. Degludec, aspart and glargine were all administered using pen-injectors, except in the USA where glargine was used in vials/syringes (participants from USA constituted 27% of the glargine sample).

### Measurement of HbA_1c_ and hypoglycaemia

HbA_1c_ was measured at clinic visits at baseline and every 4th week (five measurements in total) and assayed using a validated high-performance liquid chromatography method. Hypoglycaemia was classified as ‘confirmed’ if accompanied by a plasma glucose measurement of 3.1 mmol/l (56 mg/dl) and ‘severe’ if third-party assistance was required. Episodes were considered ‘nocturnal’ if onset occurred between 23.00 and 05.59 h, inclusive. Participants recorded details of each hypoglycaemic event in their diaries. Events were collated at each weekly visit.

### Measurement of health status

**FIGURE 1:**
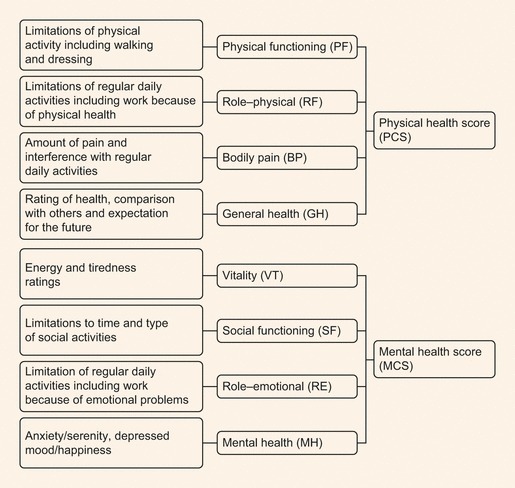
The structure of the Short Form 36 Health Survey (SF-36) instrument. Elements of physical health are assessed in four domains, and elements of mental health in another four, and summarized in a physical and mental health component score. Each domain contains two to nine separate questions.

Participants’ health status was measured at baseline and at 16 weeks using the SF-36 version 2. The SF-36 has eight domains: physical functioning, bodily pain, role—physical, general health, vitality, social functioning, role—emotional and mental health, which can be combined to give two summary component scores (Fig. 1). The first four domains give a physical component score (PCS) representing predominantly physical well-being, with the latter four providing a mental component score (MCS) comprising aspects of mental health. The questionnaire was translated and linguistically validated in all relevant languages. Participants were instructed and then left alone to complete the questionnaire.

### Statistical analyses

Treatment differences in HbA_1c_ at 16 weeks were estimated by analysis of variance (ANOVA), adjusted for country, sex, age and HbA_1c_ at randomization. Rate of hypoglycaemia was estimated by a negative binomial regression model, in which the number of episodes/patient-year of exposure (events/patient-year) was adjusted for country, sex, age and HbA_1c_ at randomization.

Changes in all eight domains of the SF-36 and physical and mental component scores were analysed by ANOVA, with treatment, country and sex as fixed effects, and age, baseline HbA_1c_ and baseline values as covariates. To investigate potential influence of the use of vials/syringes for subjects in the USA randomized to glargine, the ANOVA model allowed for country-specific treatment differences. The model would be simplified to enable an estimate of the overall treatment difference across all countries in the event that the corresponding interaction between treatment difference and country was not significant.

The SF-36 does not have a fixed minimal important difference in diabetes. However, Cohen’s effect size is noted in the SF-36 user manual as an oft-cited minimal important difference criterion [14]. Effect size is the mean change between groups divided by the baseline standard deviation. An effect size of 0.2 is considered ‘small’, 0.5 ‘moderate’ and 0.8 ‘large’.

## Results

### Clinical efficacy of degludec vs. glargine [13]

At 16 weeks, both treatment groups had achieved a comparable level of glycaemic control [HbA_1c_: degludec–glargine difference 1.1 (95% CI –1.5 to 3.7) mmol/mol [0.10 (95% CI –0.14 to 0.34) %]. Confirmed nocturnal hypoglycaemia was significantly reduced with degludec vs. glargine [5.1 vs. 12.3 events/person-year, respectively; relative rate 0.42 (95% CI 0.25–0.69)], and overall hypoglycaemia was numerically less frequent [47.9 vs. 66.2 events/person-year, respectively; relative rate 0.72 (95% CI 0.52–1.00)]. The significant difference in confirmed nocturnal hypoglycaemia rate reflected both the number of people experiencing at least one event (22% absolute difference between arms in favour of degludec) and the number of non-severe events in the study in those with at least one event (degludec 3.0 events per person, glargine 4.1 events per person).

### Health status

After 16 weeks, degludec produced a significant improvement in the mental component score of 3.01 (95% CI 0.32–5.70) vs. glargine (Table 1), with a Cohen’s effect size of 0.42, suggesting a small-to-medium clinically meaningful difference. This improvement was predominantly attributable to a significant moderate difference in the social functioning domain [8.04 (95% CI 1.89–14.18), Cohen’s effect size = 0.49] and a significant small-to-medium difference in the mental health domain [2.46 (95% CI 0.10–4.82), Cohen’s effect size = 0.39] (Table 1). The other mental component score domains were not statistically significantly changed. In all models investigated, the interaction between treatment and country was not significant (i.e. the influence of vial vs. pen in the USA did not influence the results) and therefore a common treatment difference could be estimated for all domains (Table 1).

**Table 1:** Change in Short Form 36 Health Survey (SF-36) scores at the end of the study*

SF-36 component	Degludec (*n* = 59) Mean (se)	Glargine (*n* = 59) Mean (se)	Between-arm difference (95% CI)
**Change in physical component score (PCS)**	**0.26 (1.08)**	**–0.41 (1.07)**	**0.66 (–2.30–3.62)**
Domains			
Change in physical functioning score	0.46 (1.20)	–0.69 (1.19)	1.15 (–2.14–4.43)
Change in role—physical score	1.79 (2.06)	–0.52 (2.04)	2.32 (–3.32–7.95)
Change in bodily pain score	2.11 (2.25)	0.42 (2.24)	1.69 (–4.48–7.86)
Change in general health score	–2.13 (1.06)	–0.62 (1.05)	–1.51 (–4.43–1.41)
**Change in mental component score (MCS)**	**1.88 (0.98)**	**–1.13 (0.97)**	**3.01 (0.32–5.70)**
Domains			
Change in vitality score	0.10 (1.09)	1.07 (1.08)	–0.97 (–3.95–2.01)
Change in social functioning score	5.20 (2.24)	–2.84 (2.22)	8.04 (1.89–14.18)
Change in role—emotional score	2.09 (1.84)	–0.95 (1.83)	3.04 (–2.01–8.09)
Change in mental health score	1.39 (0.86)	–1.07 (0.85)	2.46 (0.10–4.82)

* Analysed by ANOVA, with treatment, country and sex as fixed effects, and age, baseline HbA_1c_ and baseline physical and mental component score values as covariates.

In contrast to mental component score, physical component score remained similar between the insulins [+0.66 (95% CI –2.30–3.62)] and its four domains showed no significant differences between degludec and glargine (Table 1).

## Discussion

This analysis of participant responses to a health status questionnaire conducted during a 16-week investigation comparing degludec with glargine found that degludec vs. glargine therapy produced a significant improvement in one dimension of health status, represented by mental component score. This was driven by significant gains in the social functioning and mental health domains.

In contrast, neither the other domains in the mental component score (vitality and role—emotional) nor any individual dimension of the physical component score were improved. Perhaps this is unsurprising as overall blood glucose control was similar, and therefore it might be expected that the overall biochemical functioning of most organ systems would not have differed between the insulins. In any case, it is likely that differences in glucose control would have to be large or very prolonged to affect these physical domains.

The relationship between diabetes and health status is complex, and it can be difficult to speculate which aspect of the insulin therapy may explain the observed result. The treat-to-target protocol employed resulted in comparable HbA_1c_ levels, excluding glycaemic control as an explanation. A possibility is that the insulin delivery system used might affect a person’s assessment of their physical and mental status. As all participants from the USA randomized to glargine used vials/syringes, because of the unavailability of the pen injector specified in the protocol, this could potentially affect the results. However, no significant difference between treatments across the five countries included was detected, and therefore delivery system differences cannot explain the results found in this study.

The combination of fewer people experiencing at least one nocturnal hypoglycaemic event, together with the numerically lower overall (borderline significance) and statistically lower nocturnal hypoglycaemic event rates observed during the trial might explain the improved social functioning and mental health scores. Despite continued insulin therapy advances, people with diabetes still experience a substantial frequency of hypoglycaemia and it remains a limiting factor in diabetes management [15]. A recent questionnaire-based study found that people self-tested more frequently and 25% of respondents reduced their insulin dose in the days following a non-severe hypoglycaemic event. Nocturnal hypoglycaemic events resulted in 23% of respondents arriving late or missing a day of work, while one third missed a meeting or deadline [16]. A US internet-based questionnaire study found that people who self-reported low blood glucose symptoms had significantly lower mean utility scores (0.72 vs. 0.82 on the Euro-Qual Group EQ-5D measure, *P* < 0.001), indicating a lower health-related quality of life, and were significantly more likely to report increased anxiety/depression (*P* < 0.001) than people who did not experience low blood glucose symptoms in the preceding fortnight [17].

It is possible that the ultra-long action profile and lower within-person glucose variability found with degludec [11,12] may reassure users that their day-to-day glucose control is more stable, and this might improve health status. However, more work is needed to investigate the true relationship behind the observed significant health status improvement with degludec.

This study has its limitations. Participant numbers (*n* = 118), and thus statistical power, were low, so more subtle effect sizes could have been missed. However, those detected were of small-to-moderate effect size. It will be useful to see if these results can be reproduced in larger populations and in people with Type 2 diabetes mellitus. Additionally, this study was open-label, and there is a possibility that even after 16 weeks some halo effect surrounds the new insulin. Conversely, some of the participants used glargine pre-study and, for those changing to a relatively untried basal insulin preparation, anxiety could have induced increased mental burden, reducing the magnitude of the advantages identified here.

In the context of comparable overall glycaemic control to insulin glargine, insulin degludec treatment improved mental well-being as measured using the mental component score of the SF-36 questionnaire. Based on these findings, validation in a larger population during the Phase 3 investigations of insulin degludec would be advisable. Moreover, use of a larger population may allow an analysis in which it is possible to stratify SF-36 scores by presence or absence of hypoglycaemia.

## Abbreviations

SF-36: Short Form 36 Health Survey
